# Gender and Emotional Representation Matter: Own Illness Beliefs and Their Relationship to Obesity

**DOI:** 10.3389/fnut.2022.799831

**Published:** 2022-02-08

**Authors:** Carmen Henning, Stefanie Schroeder, Sabine Steins-Loeber, Joerg Wolstein

**Affiliations:** ^1^Department of Pathopsychology, University of Bamberg, Bamberg, Germany; ^2^Department of Clinical Psychology and Psychotherapy, University of Bamberg, Bamberg, Germany

**Keywords:** obesity, illness beliefs, gender, BMI, eating behavior, self-regulation

## Abstract

**Background:**

Current treatments of obesity often fail to consider gender and psychological aspects, which are essential for weight loss and weight maintenance. The aim of our study was to analyze subjective illness representations (SIRs) of adults with obesity according to the Common-Sense Self-Regulation Model (CSM) by assessing their associations with weight-related variables and gender.

**Methods:**

Data was collected via online self-assessment between April 2017 and March 2018. SIRs were operationalized by the revised Illness Perception Questionnaire (IPQ-R) and illness outcomes according to the CSM were defined as BMI, eating behaviour, physical wellbeing, bodyweight satisfaction, and shape concerns. The sample consisted of 427 adults (*M* = 42.2 years, *SD* = 10.9; 82% female) with obesity (BMI: *M* = 42.3 kg/m^2^, *SD* = 9.0). Student's *t-*tests and multiple hierarchical regression analyses were conducted with the control variables (age and BMI) and subjective illness representations and gender as independent variables.

**Results:**

The explanation of outcome variances was moderate to high (21-43%) except for restraint eating behaviour (10%). Subjective illness representations showed several significant associations with weight-related variables, especially timeline and emotional representations. Female gender was significantly associated with more restraint eating behaviour [*F*_(1, 400)_ = 4.19, *p* < 0.001] and females had unfavourable values of the weight-related variables as well as a more cyclic [*t*_(425)_ = 3.68, *p* < 0.001], and more emotional representation [*t*_(100)_ = 5.17, *p* < 0.001] of their obesity.

**Conclusion:**

The results of this study indicate that gender and subjective illness representations, especially the emotional representation, play an important role for weight-related variables. Therefore, the assessment of SIRs may constitute an economic tool to identify specific individual deficits of self-regulation.

## Introduction

In Germany, almost half of all women (47%) with a low socioeconomic status have obesity (BMI greater or equal to 30 kg/m^2^ and abnormal fat distribution) compared to 34% of the men in this group (1, 2). More than twice as many women (3%) as men (1%) suffer from obesity class III (BMI ≥ 40 kg/m^2^) (1). The male proportion in weight loss programs and obesity research is very low (3, 4). Consequently, drawing conclusions about the effectiveness of programs and influencing factors of obesity for men is difficult (5). Study results have indicated gender differences in psychosocial aspects influencing weight (6, 7). Gender-specific differences are found in the choice of food (8), diet habits (9), body dissatisfaction, and weight-related attitudes (10, 11). The latter cited study demonstrated that men with obesity were less accurate than women with obesity in their perception of weight and were less likely to try to reduce their weight. Men are more likely to participate in weight loss programs with a view to improving their professional performance or general health, whereas women tend to report social considerations and personal esteem as therapy motivation (4, 5, 12). Female gender has also been found to be a predictor of clinically meaningful weight-loss, but the effect of gender wanes in the weight maintenance phase (13, 14). The desire for sweets, craving, or stress related eating is more frequently in women (15–17). Anderson et al. (18) suggested that gender plays an important role in self-regulated behaviour such as eating behaviour and nutrition.

Women and men have been shown to differ in other self-regulation strategies such as rumination or goal-setting (19, 20). Rumination has shown to be a mediator of gender-specific differences in the strength of craving for food, binge eating, and pathological eating behaviour (21). When asked how much they would like to weigh (dream weight), female participants also had a tendency to indicate unrealistic weight-loss goals (20). Establishing realistic goals is not only part of self-regulation, it is also a predictor for better weight loss maintenance (22).

A number of existing treatment methods are offered, with the basic program usually focusing on three key elements: exercise, nutrition, and psychological therapy (23). In combination with the basic program, drug therapy can be supportive and in cases that involve comorbidities, such as type-2 diabetes or severe obesity (BMI ≥ 40), bariatric surgery is indicated. Psychological treatment interventions should focus for example on eating behaviour and should be characterised by a high degree of individualisation (24). Study results indicate that obesity is a stable condition in the long term and that individuals affected are likely either to remain obese or to become obese again after losing weight, usually because of a failure to maintain behavioural changes in the maintenance phase (25, 26). A 5% loss of the initial weight is seen as a successful treatment outcome, but only 50% of the participants of long-term weight reduction programs achieve this reduction (27). A recent study showed that to reach more than 4–6% weight loss, more intensive programs or even bariatric surgery for obesity classes I/II are required (28).

Patients with obesity in the maintenance phase need to maintain behavioural changes, even though they have already reached their desired weight (29). Consequently, the inclusion of self-regulation in treatment, especially in the maintenance phase, appears to be indispensable (30, 31). One theoretical model that considers cognitive and emotional processes of self-regulation in relation to chronic diseases is the Common-Sense Self-Regulation Model [CSM; (32, 33)]. The CSM is a parallel process model that describes individual self-regulation processes while considering a wide range of influencing variables (32–35). The CSM postulates that patients develop a subjective mental picture of their disease—the so-called subjective illness representations (SIRs) (36). SIRs are influenced by external determinants, for example, experiences associated with the illness such as medical consultations. Among other factors, these SIRs have a direct effect on illness-related outcomes (37, 38).

SIRs include several cognitive representations and one emotional representation, which relates primarily to depressive moods and anxieties (39). The cognitive dimensions include the individual perception of the course of time (chronic vs. acute), the cycle (stable vs. fast changes), and the consequences of the illness (33). Individuals with a chronic disease also tend to form subjective representations not only about their ability to control the illness (personal control) but also about their control throughout the treatment (treatment control). Both control dimensions are positively correlated to social functioning and more favourable health outcomes (38, 40). Moreover, the last supplemented representation (SIR of coherence) reflects how comprehensible and intelligible the illness is to the affected person. Previous research has indicated that threat-related representations (consequences, timeline) and a more emotional representation are negatively associated with health outcomes such as psychological wellbeing, health related quality of life, and functioning (38, 40–42). Recent evidence suggests that the activation of a chronic model of illness, representation of viewer consequences, more personal control, and lower emotional involvement indicate a more preferable outcome and course of treatment (43–46).

The CSM is applied in research and in practise, for example, to examine the self-management of patients, to develop individual intervention goals, to understand health behaviour (40, 47), to predict therapy motivation or adherence (48), or to develop theoretically based intervention programs (49, 50).

An association between SIRs and treatment outcomes has been confirmed for several chronic diseases, including asthma (51, 52), diabetes (53), cardiovascular diseases (54, 55), chronic fatigue syndrome (56), and functional somatic syndromes (57). Defined as a chronic illness, obesity is linked to several other chronic disorders, such as type-2 diabetes mellitus, cardiovascular and musculoskeletal diseases (2, 58, 59). Therefore, an empirical investigation to determine whether the CSM is also a suitable explanation and application model for obesity is a logical step. Depending on the severity of obesity, the affected people differ in health beliefs (60) and personal confidence in the ability to lose weight (61, 62). According to Lewis et al. (60), participants with severe obesity expected more serious health consequences because of their weight and reported more fear based feelings like being worried or scared than participants with moderate obesity (obesity class I and II, BMI = 30–39.9 kg/m^2^). The latter are partly unaware that they have obesity and perceive more personal control over the change in lifestyle to lose weight. Improving self-management and self-regulation has been shown to support adherence and weight maintenance, although the underlying cognitive mechanisms remain to be identified (18, 27, 29, 63). Bonsaksen et al. (44, 45) compared the SIRs of adults with severe obesity and patients with chronic obstructive pulmonary disease (COPD). The results indicate that participants with severe obesity had significantly higher representations of consequences, personal control, and emotions but significantly lower levels of treatment control and the activation of a more acute model of their illness (44). The authors demonstrated that the SIRs in individuals with severe obesity continuously changed over a year after attending a patient education course (45). Timeline, consequences, and emotional representations were reduced, and they felt more personal control. Bauer et al. (64) showed that matching the patient's attitude and the treatment methods lead to better weight loss results. Therefore, the evaluation of illness beliefs, that is, the “patient perspective” could be useful parameters for the individualisation and the success of obesity treatment (65, 66).

In summary, applying the CSM in the treatment of obesity appears to be a promising approach. To date, however, only particular subsections of the CSM or self-regulation have been investigated in obesity without differentiating gender (45, 60, 65). The aim of this study is therefore not only to transfer the CSM of self-regulation to obesity but also to focus specifically on gender differences. We investigated the direct link of SIRs to weight-related variables (dependent variables), which were defined according to previous research as BMI, shape concerns, bodyweight satisfaction, physical wellbeing, and eating behaviour.

The central research questions of this explorative study were:

1. Which expressions of the SIRs are favourable regarding weight-related variables such as physical wellbeing, bodyweight satisfaction, BMI, shape concerns, and eating behaviour?
2. How does gender affect the associations between SIRs and weight-related variables?

We hypothesised that (I) a more chronic and emotional representation as well as the feeling of more consequences are associated with worse values of weight-related variables and that (II) high control dimensions are associated with better values of weight-related variables. We hypothesised that gender is significantly associated with (IV) all links between SIRs and weight-related variables and that (V) females will show higher emotional representation.

To the best of our knowledge, no existing studies have focused on gender differences in SIRs and their relation to weight-related variables in obesity. This research therefore constitutes an innovative approach.

## Materials and Methods

### Procedure

The study was part of a larger project, which was entirely approved by the ethics committee of the University of Bamberg, Germany. The project was conducted in accordance with the Declaration of Helsinki. The Institutional Review Board of the Ruhr-University Bochum approved the project (no. 18-6415). Data collection for this cross–sectional study took place via online assessment from April, 2017 until March, 2018, with participants having the chance to win one of four €50 shopping vouchers. The link to the self-report questionnaire was public and promoted by email to encounter groups, nutrition experts, and clinical institutions, as well as by postings on social media platforms. The recruitment message stated that we were looking for evaluating psychological factors and self-perceptions of one's obesity. To avoid a systematic selection effect of a pseudo-random sample, we targeted especially male groups or associations. Consequently, the sample is disproportionately stratified concerning gender, given that the proportion of males is still less than in the population.

### Participants

**Figure 1:**
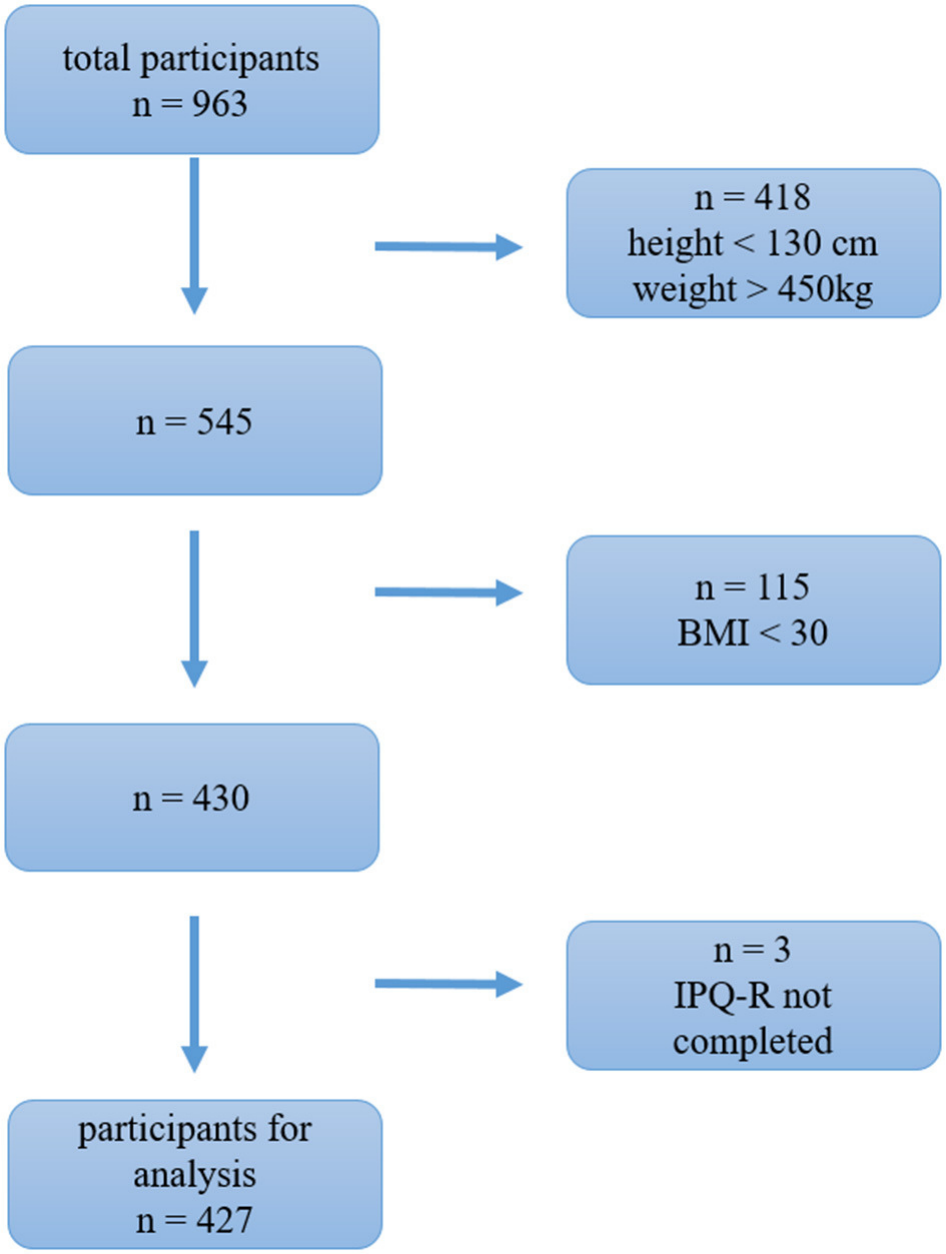
Sample conformation process.

All participants were informed about the contents of the study, and they provided written informed consent. A total of 962 participants completed the online questionnaire, and after applying the inclusion criteria (BMI ≥ 30 and finalisation of the IPQ-R questionnaire), the final sample comprised 427 participants of which 350 (82%) were female and 77 (18%) were male. The recruitment process explains the reduction of the participants. Given that the link to the questionnaire was distributed very broadly to the general public, we first omitted data from participants who provided illogical inputs (<130 cm, >450 kg) (see Figure 1 for sample conformation process). Nearly all participants were of German origin (97%) with an average age of 42.5 years (*SD* = 10.7; *Min* = 21; *Max* = 76), whereby the male sample was significantly older (males: *M* = 49.2 years; females: *M* = 41.0 years; see Table 1). The BMI was calculated by using self-reported weight and height and was on average 41.7 (*SD* = 9.0; Min = 30.0; Max = 83.6). The participants were affected by either obesity class I (BMI = 30–34.9; *n* = 112), class II (BMI = 35-39.9; *n* = 93), or class III (BMI ≥ 40; *n* = 222). Table 1 shows that male's BMI (*Mdn* = 34.0; *Min* = 30.2; *Max* = 77.2; BMI ≥ 40: 27.3 %) and female's BMI (*Mdn* = 41.8; *Min* = 30.0; *Max* = 83.6; BMI ≥ 40: 57.4 %) differed significantly, *t*_(425)_ = 4.10; *p* < 0.001; |*d*| = 0.52. Females had higher BMIs, such that the average of the female sample was in the obesity class III.

**Table 1:** Descriptive results and *t*-test parameters for gender differences.

**Scale**	**Total**		**Female**		**Male**						
	***n***	***M* (SD)**	***N***	***M* (SD)**	***n***	***M* (SD)**	***t***	**df**	***p***	**CI**	**IdI**
**SIRs**
Timeline	427	3.78 (0.77)	350	3.81 (0.78)	77	3.65 (0.74)	1.61	425	0.108	(−0.15; 0.46)	0.21
Cyclic	427	2.89 (0.77)	350	2.96 (0.77)	77	2.60 (0.71)	3.68	425	<0.001^***^	(0.05; 0.65)	0.47
Consequences	427	3.62 (0.85)	350	3.67 (0.84)	77	3.40 (0.85)	2.52	425	0.012	(−0.06; 0.60)	0.32
Personal control	427	3.88 (0.75)	350	3.83 (0.76)	77	4.11 (0.65)	−2.97	425	0.003	(−0.57; 0.01)	0.38
Treatment control	427	3.58 (0.69)	350	3.58 (0.69)	77	3.58 (0.69)	−0.05	425	0.963	(−0.27; 0.27)	0
Coherence	427	3.93 (0.91)	350	3.94 (0.67)	77	3.90 (0.78)	0.46	425	0.649	(−0.23; 0.31)	0.06
Emotional representation^a^	427	3.95 (0.92)	350	4.07 (0.85)	77	3.42 (1.02)	5.17	100	<0.001^***^	(0.25; 1.04)	0.74
**Weight-related variables**
BMI	427	41.69 (9.05)	350	42.52 (8.66)	77	37.93 (9.85)	4.10	425	<0.001^***^	(1.11; 8.06)	0.52
Bodyweight satisfaction	388	21.49 (24.76)	319	19.85 (23.85)	69	29.09 (27.51)	2.84	386	0.005	(−19.37; 0.90)	0.38
Shape concerns	397	33.31 (11.26)	328	34.66 (10.83)	69	26.87 (11.09)	5.41	395	<0.001^***^	(3.31; 12.27)	0.72
Eating behaviour
- Restraint	411	28.52 (6.81)	336	29.14 (6.64)	75	25.71 (6.91)	4.02	409	<0.001^***^	(0.78; 6.09)	0.51
- Emotional	411	31.08 (9.97)	336	32.32 (9.48)	75	25.53 (10.28)	5.51	409	<0.001^***^	(2.96; 10.61)	0.71
- External	411	32.05 (7.88)	336	32.81 (7.67)	75	28.67 (7.98)	4.20	409	<0.001^***^	(1.07; 7.21)	0.54
Low physical wellbeing	395	3.67 (.95)	327	3.73 (.95)	68	3.37 (.90)	2.84	393	0.005	(−0.03; 0.75)	0.38
**Other**
Age	427	42.49 (10.72)	350	41.02 (10.35)	77	49.18 (9.83)	−6.32	425	<0.001^***^	(−12.17; −4.14)	0.80
PHQ-9	386	10.80 (6.38)	320	11.35 (6.30)	66	8.14 (6.12)	3.79	384	<0.001^***^	(0.57; 5.85)	0.51

a *Welch correction; p-value (two-sided), significance at p < 0.002 (see Bonferroni adjustment)*.

*** *p < 0.001*.

### Instruments

#### Demographic Variables

We assessed the demographic variables such as age and gender at the beginning of the questionnaire. The screening for *depression* was made with the Brief Patient Health Questionnaire (PHQ-9 (67, 68); α = 0.89), which assesses the prevalence of depression symptoms in the last 2 weeks on a 4-point scale (0 = *never* to 3 = *nearly every day*). The cut-off value was 10 according to the manual. Scores ≥10 are equivalent to a positive screening for depressive symptoms.

#### Subjective Illness Representations

The subjective illness representations were assessed using the German version of the revised Illness Perception Questionnaire [IPQ-R; (39, 69)]. The analysis included seven subscales measuring different SIRs that refer to the CSM: timeline acute/chronic (five items; Cronbach's α = 0.82 of sample in this study), cyclic (four items; α = 0.66), consequences (five items; α = 0.81), personal control (four items; α = 0.78), treatment control (four items; α = 0.65), illness coherence (five items; α = 0.73), and emotional representation (five items; α = 0.89). The answering format was a 5-point scale from *strongly disagree* to *strongly agree*. A more chronic model of obesity is represented by high scores on the chronic timeline scale, meaning that the individual expected a longer course of the disease. High scores for cyclic representations represent a mental image of unsteady progress with numerous ups and downs in the progression of the illness.

High results for consequences and emotional representations indicate that individuals with obesity associate their illness with many or severe consequences or with negative feelings like shame or guilt. The coherence scale demonstrates that the illness is more coherent and makes sense to the patients. For both control dimensions, high scores show that participants represent their illness on a manageable level, and they feel able to control the illness without help (personal) or with a physician's help (treatment).

#### Dependent Variables: Weight-Related Variables

In addition to BMI, we analysed bodyweight satisfaction, shape concerns (70, 71), eating behaviour (72), and physical wellbeing (73, 74), whereby BMI was assessed by self-reported height and weight.

*Bodyweight satisfaction* was assessed by using a bipolar rating scale with a slider question type (“How satisfied are you with respect to your bodyweight in general?”; 0 = *very unsatisfied*, 100 = *very satisfied*).

Subjective *concerns about body shape* (within the last 28 days) were assessed by using the mean of the 8 item subscale “Shape Concern Scale” of the Eating Disorders Examination-Questionnaire [EDE-Q; α = 0.86; (75, 76)]. The attitudinal items (e.g., “*Have you had a definite desire to have a totally flat stomach?*”) were rated using a 7-point format (0 = *characteristic was not present*; 6 = *characteristic was present every day or extremely present*) by which higher scores imply greater shape concerns.

*Eating behaviour* was examined using the total score of the German version of the Dutch Eating Behaviour Questionnaire [DEBQ; (77, 78)]. The 30-item (α = 0.91) DEBQ assesses three eating behaviour domains (10 items each): r*estraint eating* (α = 0.86; e.g., “I deliberately eat less in order not to become heavier.”), *external eating* (α = 0.90; e.g., “I eat more than usually when I see others eating.”), and *emotional* eating (α = 0.94; e.g., “When I'm irritated, I have a desire to eat.”). The items are formulated as statements and are rated on a 5-point Likert scale (1 = *never*; 5 = *very often*) by which higher scores stand for worse eating behaviour.

*Physical wellbeing* (PW) was assessed using a German standardised questionnaire with the mean of the total scale (79). The FEW-16 measures habitual physical wellbeing based on the WHO health definition and Antonovsky's concept of salutogenesis (80). It operationalizes the positive pole of the health-disease continuum and represents the subjective component of health. The original answering format of the four statements for each subscale was a 6-point Likert scale (0 = *does not apply at all* to 5 = *fully applies*). We recoded the items so that a higher score represents low physical wellbeing (i.e., 5 = *does not apply at all*).

#### Statistical Analysis

Three-step hierarchical multiple regressions were conducted to assess the extent that the SIRs and gender add predictive value to the weight-related variables. This statistical procedure was used to answer the research questions. (1) Which expression of the SIRs are favourable in regard to the weight-related variables? (2) Does gender moderate these associations? Given that age has been analysed as a weight loss predictor in previous studies, we controlled also for age in addition to BMI (81, 82). Both variables were entered in the first step to control for these parameters in all models except the model with BMI as dependent variable (only controlled for age in this model). The SIRs were added as independent variables at the second step, followed by gender at stage three (encoding: 0 = female, 1 = male). Gender was added as a separate step to assess if this variable changed the explanation of the weight-related variables significantly in addition to the SIRs. A multiple regression was run for each of the six dependent variables. The significance level was set at *p* < 0.05 and was maintained through a Bonferroni correction for multiple testing (16 *t*-tests and seven hierarchical regressions: *p* < 0.002). The partial regression plots and a plot of studentized residuals against the predicted values indicated linearity. The independence of residuals was assessed by Durbin-Watson values (between 1.81 and 2.11). We found no evidence of multicollinearity based on tolerance values (>0.10) and correlations (<0.70) that were all within accepted limits. The few outliers (±3 standard deviations) that were found seemed to be logical scores, and given that leverage values (<0.200) and values for Cook's distance (<1) were accepted, no outliers were deleted from the data. The assumption of normality was not met for bodyweight satisfaction and shape concern (assessed by Q-Q Plot), but this deviation was negligible because of our sample size. After checking for homogeneity by using a plot of studentized residuals vs. unstandardized predicted values, the regression analyses with body satisfaction, shape concern, and BMI as dependent variables were conducted with bootstrapping (2,000 simulations, BCa, 99.8 % confidence interval).

Comparisons between males and females were performed by student's *t*-tests, and homogeneity of variance was checked with Levene's test. The Welch test was conducted for the emotional representations because of the heterogeneity of the variances. Hedge's g was calculated for effect sizes. The common abbreviation |d| was used, with |d| < 0.2 indicating a small, |d| < 0.5 a medium, and |d| < 0.08 a large effect. The dataset contained missing values for some scales up to 9.6% of the participants who completed the IPQ-R (39) but not the remaining questionnaire. The MCAR test according to Little was not significant (*Chi*^2^ = 0; *df* = 9; *p* = 1). Thus, the values were missing completely at random (MCAR). Given that only dependent variables had missing values, an imputation of the missing values was not necessary and a listwise exclusion was performed for the regression analysis, as set by default in SPSS [see (83)]. All analyses were conducted using SPSS version 26.

## Results

### Gender Differences

**Figure 2:**
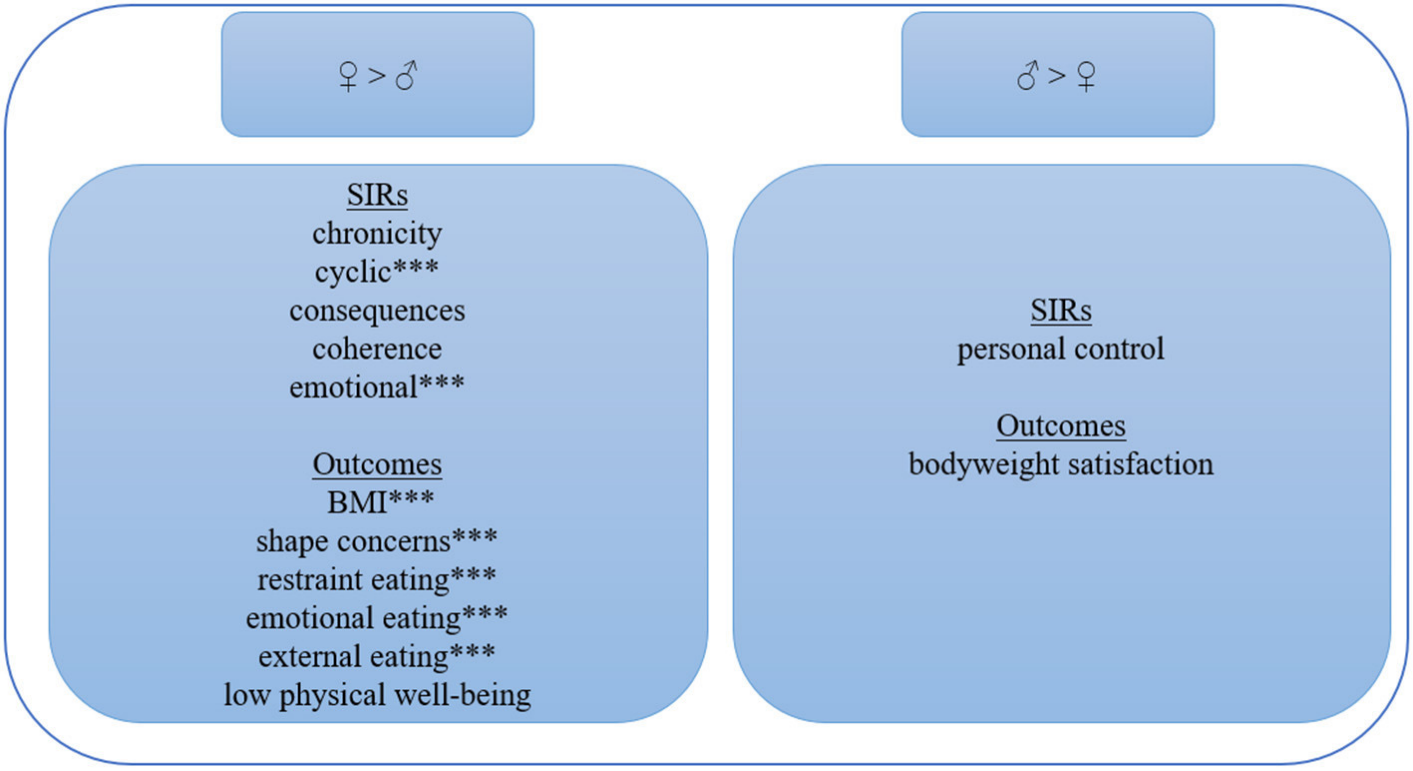
Gender differences. ***p < 0.001.

The results obtained from the *t*-tests for gender differences are presented in Table 1 and are summarised in Figure 2. Male participants perceived their obesity significantly more consistently (*IdI* = 0.47) and the emotional representation was significantly more favourable (*IdI* = 0.74). This result means that women reported more negative emotions, such as anger, worry, fear, or depression, associated with their obesity than men. No significant gender differences were found in the other dimensions of the IPQ-R: timeline (*IdI* = 0.21), consequences (*IdI* = 0.32), coherence (*IdI* = 0.06), personal (*IdI* = 0.38), and treatment control (*IdI* = 0).

All significant differences in weight-related variables were more favourably pronounced for men (*IdI* = 0.38–0.72), with shape concern (*IdI* = 0.72) and emotional eating behaviour (*IdI* = 0.71) having the greatest effect. This indicates that men had significantly lower BMI (*IdI* = 0.52), less restraint (*IdI* = 0.51), or external (*IdI* = 0.54) eating behaviour. No significant gender differences were revealed for bodyweight satisfaction (*IdI* =.38) or physical wellbeing (*IdI* = 0.38, see details in Table 1). Men were significantly older (*IdI* = 0.80) and showed no depressive symptoms, whereas women generally exceeded the cut-off value of PHQ-9 (*IdI* = 0.51).

### Hierarchical Regression Analyses

Age had a significant effect only on external eating behaviour (all data in Appendix A). In the first step, BMI was significantly associated with physical wellbeing, shape concerns, and bodyweight satisfaction. Only the latter remained significant in step 3, that is, the effect between BMI and shape concerns and physical wellbeing, respectively, became non-significant. Table 2 presents the summary of the results. The proportion of variance explained was highest for shape concerns (43%) and physical wellbeing (33%). All other proportions were classified as moderate or high, except for restraint eating (10%). The link between the dependent variables and the emotional representation of obesity was most often significant. The cyclic representation added no significant contribution to the regression equations. Adding gender in stage three revealed significant changes in explained variance for shape concerns and all scales of eating behaviour. Gender was significantly associated with restraint and emotional eating behaviour but not external eating behaviour. Restraint eating was the only dependent variable for which the SIRs accounted for none of the variance in scores.

**Table 2:** Results of hierarchical regression analyses.

**Dependent variable**	**Step 1** **Age, BMI**		**Step 2** **SIRs**		**Step 3** **Gender**		
	***R* ^2^**	***p*** **(*R*^2^ change)**	***R* ^2^**	***p*** **(*R*^2^ change)**	***R* ^2^**	***p*** **(R^2^ change)**	**Significant link to dependent variables**
Bodyweight satisfaction	0.09	<0.001^***^	0.21	<0.001^***^	0.21	0.77	BMI (–) Emotional SIR (–)
Less wellbeing	0.05	<0.001^***^	0.33	<0.001^***^	0.33	0.76	consequences SIR (+) Treatment control SIR (–) Emotional SIR (+)
Shape concerns	0.05	<0.001^***^	0.41	<0.001^***^	0.43	0.001^***^	Emotional SIR (+)
BMI	0.02	0.002^**^	0.24	<0.001^***^	0.25	0.05	Timeline SIR (+) Consequences SIR (+)
Restraint eating	0	0.695	0.06	0.001^**^	0.10	<0.001^***^	Gender (–)
Emotional eating	0.02	0.007	0.21	<0.001^***^	0.24	<0.001^***^	Timeline SIR (+) Personal control SIR (+) Coherence SIR (+) Emotional SIR (+) Gender (–)
External eating	0.05	<0.001^***^	0.21	<0.001^***^	0.23	<0.001^***^	Timeline SIR (+) Personal control SIR (+) Emotional SIR (+) Age (–)

*p-value (two-sided), significance at p < 0.002 (Bonferroni adjustment)*.

** *p < 0.002*;

*** *p < 0.001*.

In detail, obesity perceived as chronic was associated with a higher BMI and increased emotional and external eating behaviours. An increased perception of consequences had the strongest connection to a high level of BMI and low physical wellbeing. The perception of coherence showed the only significant association with increased emotional eating behaviour. Perceived personal control accounted for significant variance in external eating behaviour, and the representation of high treatment control accounted for significant variance in better wellbeing. Finally, a more pronounced emotional representation contributed significantly to less body satisfaction, more shape concerns, increased emotional, and external eating behaviour, as well as less physical wellbeing. Interestingly, the emotional representation had no significant association with the BMI level. Female gender accounted for significant variance in emotional and (as the only independent variable) for restraint eating.

Overall, these results indicate not only gender differences in SIRs and in weight-related variables but also that most of the SIRs, especially the timeline and emotional representation, explain a moderate to high amount of variance in weight-related scales.

## Discussion

To the best of our knowledge, this study is the first to systematically examine gender differences in the effect of SIRs of individuals with obesity on weight-related variables according to the CSM. Specifically, we analysed the relationships between the cognitive SIRs (timeline, chronicity, coherence, control) and emotional SIRs with shape concern, bodyweight satisfaction, BMI, physical wellbeing, and eating behaviour. Especially notable is the comparatively high proportion of male participants in our sample, which is why our study stands out from other studies in this area of research. One of the main goals of the current study was to determine the favourable expressions of the SIRs in participants with obesity. The results of this study indicate that low representations of emotions, consequences, timeline, coherence, and personal control as well as a high representation of treatment control were associated with more favourable weight-related variables. Accordingly, female participants had worse SIRs than men, and female gender was associated with worse emotional and restraint-eating behaviour. Interestingly, after adding SIRs and gender to the regression models, the level of BMI and age had no additional effects on weight-related variables.

### Gender

Females reported significantly more cyclic and as hypothesised (V) emotional representations of their obesity, but female gender only accounted for significant variance in the two eating behaviour scales (hypothesis IV rejected), thus again underlining the importance of the emotional representation of one's obesity. This result for the gender difference of the cyclic representation has been reported in the literature. For example, women tend to show more weight-cycling and go on more diets, and their craving for food depends on their female hormone cycle (84, 85). Our findings revealed, however, that shape concerns is independent of gender, which is not consistent with previous research showing that gender significantly predicted shape concerns (86).

Female gender accounted for significant variance in emotional eating and restraint eating behaviour, which is consistent with other studies (8, 87). A possible explanation is that emotional distress can interrupt the restrictive cognitive control, which regulates restraint eating (78). Restraint eating implies that individuals suppress their feelings through cognitive control. When the control is interrupted, they may give in to their feelings and overeat (87). Although females are more vulnerable to overeating because of emotional stress, they are also successful in restraint eating. Hence, they should be sensitised for the interaction between these two eating behaviours. Given that restraint eating behaviour is associated with successful weight maintenance after weight loss (88), stabilising favourable eating behaviours and enhancing strategies to better cope with negative emotions is important, especially for women. The strong link between female gender and the emotional representation of their obesity, which in turn explained the variance of most of the maladaptive weight-related variables, seems to be a crucial aspect of SIR-based treatments in obesity. The high emotional representation observed in women was supported by existing research. Studies on the causal attributions of obesity have shown that women name more emotional aspects, like family worries, as causes than men (89) and that men were more likely to consider their personality as a reason for suffering from obesity (72). These results and the findings from the current study may shed light on the gender effect in emotional representations, but they also underscore the strong bi-directional association of affective disorders like depression and obesity for women (90, 91). According to Glattacker et al. (69), emotional representation is highly correlated with anxiety, depression, and low self-efficacy. A strong association between depressive symptoms and obesity was also evident in our sample, particularly in women.

Although our female sample had a significantly higher BMI, male's and female's representations of their obesity as chronic were the same. This result could explain the unrealistic goal-setting for weight-loss in women (20) who may assume that their obesity is just a short, temporary condition and can be regulated quickly.

Our suggestion based on the current results and the study of (92) is that male participants show better weight-related variables, such as shape concerns or bodyweight satisfaction, and have more preferable SIRs as a defence mechanism. They often fail to recognise their high bodyweight as a serious illness, or they think that obesity is a “female illness.” In contrast, cardiovascular diseases are recognised as more masculine (93), which in turn influences the motivation for treatment and participation in research. Therefore, public campaigns and media should sensitise males to the seriousness of obesity and the importance of weight-loss in the prevention of further serious health consequences.

### Timeline Representation: Chronic vs. Acute

Our findings indicate that a more chronic representation is more prevalent in women with severe obesity and is more likely to affect individuals prone to emotional and external eating behaviour. This association between increased timeline representation and maladaptive outcomes is in line with our hypothesis (I) and other studies reporting on chronic diseases and BMI levels (37, 94). Moreover, this group of patients with an increased timeline representation tend to follow more diets (85) and are less confident of losing weight (61). In contrast, a more chronic view did not predict poorer physical wellbeing in studies investigating other diseases (37). Consequently, a more chronic representation of obesity can be considered disadvantageous because of its association with maladaptive weight-related variables. However, existing research has also reported that the activation of a less chronic model is a key problem in terms of the self-management of chronic illnesses in general and specifically regarding obesity (35, 95). The current study found no support for this view. A possible explanation for this inconsistency might be that the course of obesity or the phase of treatment (weight loss vs. maintenance) determine which occurrence of timeline representation is favourable. This view was supported by the link between SIRs and motivational processes for individuals with eating disorders (96). Our suggestion is that whether or not it is advantageous to see obesity as more acute than chronic depends on the stage of therapy. Therefore, an investigation of the patient's timeline representation as well as cognitive preparation for weight loss and a discussion of relapse should form part of behavioural therapy in obesity (97).

### Cyclic Representation

Existing research emphasises that representations about the cyclic character of illness are associated with the maintenance level of motivation for eating disorders (96) and could therefore also be important for motivational aspects relating to obesity. However, the current study found no support for a relationship between the cyclic representation of obesity and the examined outcome variables. In contrast to a study with patients having cardiovascular diseases and overweight/obesity, no evidence was found that a more cyclic view explain variances of the BMI (94).

### Consequence Representation

Studies on other chronic diseases have revealed a strong link between the subjective representation of consequences and disease outcomes, as well as a negative correlation between perceived consequences and maladaptive outcomes such as poorer physical quality of life (37, 38, 41). Our results for obesity are consistent with these findings and our hypothesis (I). Highly pronounced consequential representations was significantly associated with a high BMI and worse physical wellbeing, whereby the representations of consequences showed the strongest link to the BMI compared to the other SIRs. This result is consistent with the finding of Dempster et al. (46).

A possible explanation for the relationship is that higher weight is associated with more comorbidities (98, 99), risk for other diseases such as diabetes mellitus (2), and difficulties with activities, mobility, or daily life (100, 101). The sensitisation to the fact that patients consider their obesity as the cause of these consequences can motivate them to lose weight and to maintain that loss. Our findings indicate that this could lead to an improvement in their physical wellbeing such as resilience, vitality, and inner peace. We hypothesise that to evaluate and track not only the actual physical and psychological consequences but also the representation of those consequences may be beneficial. These representations can be used to show progress in psychological treatment economically without the need for detailing medical reports but instead with the inclusion of the patient's perspective. However, contrary to our hypotheses there was no significant association to bodyweight satisfaction, shape concern, or any eating style.

### Personal Control Representation

A high representation of personal control explained the variation in more emotional and external eating behaviour. We suggest that individuals with a high personal control representation are more sensitive to food cues such as the smell of food or an obesogenic environment, for example, passing by a café or restaurant (78, 87). The participants need to have a high personal control to resist these cues. The conclusion that low personal control could be more favourable in obesity is in contrast to our hypotheses (III) and other study results (45, 62). The detrimental effect of high personal control leading to more external eating behaviour may seem counter intuitive. One explanation could be that participants not only perceive personal control but also responsibility or even blame, which could explain the increased emotional eating behaviour. An alternative explanation could be that the representation of high personal control does not necessarily lead the individual to derive action from it. Increased external eating behaviour serves as a mediator of the relationship between impulsivity and increased food intake (102). We suggest that perceived personal control is not related to active personal control. Thus, this representation requires more research and a clear distinction from personal blame, which may inhibit intervention and action.

### Treatment Control Representation

The treatment control scale was only significant for higher levels of physical wellbeing. This finding confirms the link between treatment control and positive health outcomes, which was found in previous research and supports our hypothesis (III) (40). We suggest that patients with a high treatment control representation use the support of professionals, which is also an adaptive coping strategy. Therefore, a high treatment control representation can be seen as a protective factor for individuals with obesity.

### Coherence Representation

Our results indicate that emotional eating behaviour is especially associated with a high sense of coherence, which could be a coping strategy to regulate emotions (87). Moreover, reducing emotional eating seems to be one of the self-regulation mediators for weight loss in females (103). It should be noted that the coherence scale of the IPQ-R directly assesses the disease and not the general sense of coherence (SOC). The general SOC is negatively associated with BMI, and women with obesity generally have less SOC than men (104). However, the current study found no support for these relationships in individuals with obesity. The representation of coherence showed no significant link to the BMI, and we found no gender differences. Our assumption is that a high degree of coherence representation indicates a feeling of personal responsibility for obesity, which can also be a negative feeling or internalisation of blame. Bonsaksen et al. (45) suggested that a high level of coherence and understanding for obesity does not lead to the initiation of weight-loss. They also suggested that patients think they already know everything about the reasons for their obesity. Of the weight-related variables that we investigated and in contrast to findings for other chronic diseases (37), a low representation of coherence can be considered favourable for obesity regardless of gender. Our suggestion is that obesity is a more emotional illness than other chronic diseases and that most of the affected individuals live in a vicious circle. The high representation of coherence might be a trigger for emotional stress, which in turn can lead to maladaptive coping strategies such as emotional eating.

### Emotional Representation

The emotional representation was, hypotheses conform (I), the most consistent, significant correlate of the dependent variables such as less bodyweight satisfaction, less physical wellbeing, more shape concerns, and increased emotional and external eating. The latter has been shown to mediate the relationship of impulsivity and food intake (102). The link between self-regulated behaviour, such as dieting, has not been detected in other research (47). It is somewhat surprising that adding SIRs to the regression model in our study eliminated the link to BMI, especially in outcomes for which higher weight would seem to be an obvious contributor. Thus, the emotional representation but not BMI was significantly associated with, for example, shape concern and physical wellbeing. This representation showed the only significant association with bodyweight satisfaction, which underscores that the mental picture of the illness could be much more important for weight-related variables than the actual weight. We suggest that emotion regulation skills training could be a key element in improving the subjective emotional representations and associated weight-related variables, as well as psychoeducational training, which can reduce emotional stress (45). In summary, the integration of emotional representation is essential in obesity therapy, especially for women.

The results of this study indicate that low representations of emotions, consequences, coherence, and personal control as well as a high representation of treatment control are associated with more favourable weight-related variables. Accordingly, female participants had worse SIRs than men, and female gender was associated with worse emotional and restraint eating behaviour.

### Limitations

This study is based on a cross-sectional survey, and therefore no causal conclusions can be drawn. Given that no systematic investigation of SIRs with regard to gender and weight-related variables had previously been undertaken for obesity and that men in particular are under represented as participants in this field of research, obtaining a large sample was important to be able to generalise the results. Such cross-sectional designs concerning the CSM have already been conducted for other chronic diseases (53, 94). Given our non-clinical sample, obesity was defined based on BMI, which was calculated from self-reported weight and height. For reasons of economy, this is quite usual in this field of research. Nevertheless, self-reported height and weight is subject to distortions to the effect that women in particular may have a lower BMI in reality (105, 106). Some researchers emphasise that this may not be a gender issue but that under- and over-reporting depends on BMI level and eating behaviour (107). More than half of the sample in this study were affected by obesity class III, and at this obesity level, BMI is more likely to be underestimated (108). Therefore, our results should be particularly applicable to the target group of participants with obesity.

Some aspects, which were not considered because of the focus on gender, could play a role in the association of SIRs and weight-related variables. Previous research, for example, has indicated that the education level of the participants can be a moderator of the link between obesity and cognitive mechanisms (109, 110). Eating disorders, such as binge-eating disorder, could also affect the cognitive representations and their associations, which requires further investigation (60, 111).

## Conclusion

This study lends further support to previous research findings (18) to the effect that self-regulation, SIRs, and especially gender play an important role in weight-related variables. The results emphasise the cognitive and emotional aspects of eating behaviour, physical wellbeing, shape concerns, and bodyweight (satisfaction), and they provide evidence for the inclusion of SIRs in interventions for individuals with obesity. The assessment of SIRs may constitute an economic tool in treatment planning and control in obesity to identify specific cognitive and emotional individual deficits of self-regulation. Based on our results, we recommend to evaluate the SIRs to ensure that the treatment methods match the mental picture of the patient and—more importantly—to use the SIRs for targeting program contents to achieve better weight-loss (maintenance) and a higher proportion of males.

The results indicate that the CSM and its assumptions can be adapted to obesity and that the integration of this model is promising for an individual- and gender-sensitive therapy. Particular attention should be paid to cognitive representations of timeline and consequences, as well as the emotional representation of obesity. Ultimately, gender-sensitive interventions should be developed, especially in the treatment of pathological eating behaviour in females. Males should be made more aware of their obesity and the physical and psychological problems it causes to increase their willingness to undergo therapy. In addition, an all-encompassing examination of the CSM components including the self-system and the illness stimuli for obesity is recommended for the two different phases (weight reduction and maintenance) of treatment.

## Data Availability Statement

The raw data supporting the conclusions of this article will be made available by the authors, without undue reservation.

## Ethics Statement

The studies involving human participants were reviewed and approved by Ethic Committee of the University of Bamberg. The patients/participants provided their written informed consent to participate in this study.

## Author Contributions

CH, SS, SS-L, and JW contributed to conception and design of the study. SS designed the first draft of the questionnaire. CH organised the database, performed the statistical analysis, and wrote the first draft of the manuscript. All authors contributed to manuscript revision, read, and approved the submitted version.

## Funding

This work was supported by the German Federal Ministry of Education and Research (BMBF Grant Number. 01GL1719A).

## Conflict of Interest

The authors declare that the research was conducted in the absence of any commercial or financial relationships that could be construed as a potential conflict of interest.

## Publisher's Note

All claims expressed in this article are solely those of the authors and do not necessarily represent those of their affiliated organizations, or those of the publisher, the editors and the reviewers. Any product that may be evaluated in this article, or claim that may be made by its manufacturer, is not guaranteed or endorsed by the publisher.
