# Supplementary material for: Gender and Emotional Representation Matter: Own Illness Beliefs and Their Relationship to Obesity
Source: Front Nutr. 2022 Feb 8;9:799831. doi: 10.3389/fnut.2022.799831 (PMC8863172; doi:10.3389/fnut.2022.799831)
Supplement: Supplementary file 1 [file Data_Sheet_1.PDF]

Appendix A

Summary of Hierarchical Regression Analysis for variables associated with ***Bodyweight Satisfaction****.*

Variable	B	SE[1]	BCa[1]	p[1]	β	t	p	R	R^2	ΔR^2	F (df1,df2)	p (F change)
**Step 1 (control variables)**								.29	.09	.09*	18.28 (2,385)	<.001
Age	.08	.11[a]	[-.25;.45][a]	.500[a]	.04	.72	.475					
BMI	-.78	.15[a]	[-1.22;-.39][a]	.001[a]	-.29	-5.80*	<.001					
**Step 2 (SIRs)**								.46	.21	.12*	11.04 (7,378)	<.001
Age	-.01	.11[a]	[-.35;.36][b]	.923[a]	-.01	-.11	.913					
BMI	-.55	.16[a]	[-1.01;-.07] [a]	.001[a]	-.20	-3.84*	<.001					
Timeline / Chronicity	-3.09	1.86[a]	[-8.89;3.25][a]	.097[a]	-.10	-1.83	.067					
Cycle	2.04	1.74[a]	[-2.73; 7.03][a]	.238[a]	.07	1.22	.223					
Consequences	1.02	1.60[a]	[-3.65;5.64][a]	.526[a]	0.35	.59	.559					
Personal Control	-1.29	1.83[a]	[-6.76;4.59][a]	.474[a]	-.04	-.71	.476					
Treatment Control	4.40	1.97[a]	[-1.73;10.69][a]	.027[a]	.13	2.41	.016					
Coherence	-.44	1.90[a]	[-7.23;5.39][a]	.824[a]	-.01	-.24	.814					
Emotional Representation	-9.52	1.68[a]	[-15.56;-3.75][a]	.001[a]	-.35	-5.88*	<.001					
**Step 3 (Gender)**								.46	.21	0	9.92 (1,377)	.769
Age	-.02	.12[b]	[-.37;.43][b]	.874[b]	-.01	-.17	.863					
BMI	-.55	.16	[-1.08;-.04][b]	.001[b]	-.20	-3.77*	<.001					
Timeline / Chronicity	-3.08	1.87[b]	[-8.96;3.17][b]	.096[b]	-.10	-1.83	.068					
Cycle	2.09	1.80[b]	[-2.92;7.15][b]	.235[b]	.07	1.25	.214					
Consequences	.97	1.63[b]	[-3.70;5.77][b]	.559[b]	.03	.55	.583					
Personal Control	-1.36	1.87[b]	[-7.00;4.63][b]	.464[b]	-.04	-.74	.457					
Treatment Control	4.42	1.99[b]	[-1.72;10.64][b]	.025[b]	.13	2.41	.016					
Coherence	-.39	1.89[b]	[-7.20;5.58][b]	.849[b]	-.01	-.21	.834					
Emotional Representation	-9.43	1.67[b]	[-15.48;-3.90][b]	.001[b]	-.35	-5.74*	<.001					
Gender	.97	4.21[b]	[-11.47;14.52][b]	.815[b]	.02	.29	.769					

Note. $N = 388$, * = significant ($p < .002$; see Bonferroni adjustment); BCA 99.8% CI; [1]values after bootstrapping; [a]based on 1,473 samples; [b]based on 1,472 samples

Summary of Hierarchical Regression Analysis for variables associated with ***low Physical Well-Being****.*

Variable	B	SE	β	t	p	R	R²	ΔR²	F (df1,df2)	p (F change)
**Step 1 (control variables)**						.23	.05	.05*	10.43 (2;392)	<.001
Age	-.01	.00	-.05	-1.08	.280					
BMI	.02	.01	.21	4.24*	<.001					
**Step 2 (SIRs)**						.58	.33	.28*	21.11 (7;385)	<.001
Age	-.00	.00	-.01	-.20	.844					
BMI	0	.01	-.01	-.05	.961					
Timeline / Chronicity	.16	.06	.13	2.67	.008					
Cycle	.11	.06	.10	1.92	.055					
Consequences	.24	.06	.21	3.86*	<.001					
Personal Control	-.13	.06	-.10	-1.99	.048					
Treatment Control	-.24	.06	-.18	-3.71*	<.001					
Coherence	.13	.06	.09	2.02	.044					
Emotional Representation	.26	.06	.25	4.68*	<.001					
**Step 3 (Gender)**						.58	.33	0	18.97 (1;384)	.759
Age	0	.00	-.01	-.12	.908					
BMI	0	.01	-.00	-.08	.939					
Timeline / Chronicity	.16	.06	.13	2.65	.008					
Cycle	.11	.06	.09	1.89	.059					
Consequences	.24	.06	.21	3.87*	<.001					
Personal Control	-.12	.06	-.10	-1.94	0.53					
Treatment Control	-.24	.07	-.18	-3.70*	<.001					
Coherence	.13	.06	.09	1.98	.049					
Emotional Representation	.26	.06	.25	4.57*	<.001					
Gender	-.04	.11	-.01	-.31	.759					

Note. N = 395, * = significant ($p < .002$; see Bonferroni adjustment)

Summary of Hierarchical Regression Analysis for variables associated with ***Shape Concerns****.*

Variable	B	SE[1]	BCa[1]	p[1]	β	t	p	R	R²	ΔR²	F (df1,df2)	p (F change)
**Step 1 (control variables)**								.22	.05	.05*	9.98 (2,394)	<.001
Age	-.11	.06[a]	[-.28;.04][a]	.047[a]	-.11	-2.15	.032					
BMI	.22	.06[a]	[.04;.44][a]	<.001[a]	.18	3.57*	<.001					
**Step 2 (SIRs)**								.64	.41	.37*	30.24 (7,387)	<.001
Age	-.01	.05[a]	[-.19;.13][a]	.856[a]	-.01	-.21	.833					
BMI	-.02	.05[a]	[-.16;.16][a]	.740[a]	-.01	-.29	.771					
Timeline / Chronicity	.60	.70[a]	[-1.67;2.63][a]	.396[a]	.04	.91	.364					
Cycle	1.01	.67[a]	[-1.14;3.18][a]	.140[a]	.07	1.55	.121					
Consequences	1.74	.78[a]	[-.51;4.11][a]	.026[a]	.13	2.57	.011					
Personal Control	.91	.71[a]	[-1.28;3.41][a]	.190[a]	.06	1.32	.189					
Treatment Control	-.43	.76[a]	[-2.77;2.09][a]	.560[a]	-.03	-.61	.545					
Coherence	-.03	.74[a]	[-2.44;2.21][a]	.964[a]	-.00	-.04	.965					
Emotional Representation	6.46	.63[a]	[4.34;8.48][a]	<.001[a]	.52	10.38*	<.001					
**Step 3 (Gender)**								.66	.43	.02*	28.93 (1,386)	.001
Age	.03	.05[b]	[-.14;.18][a]	.601[a]	.03	.61	.545					
BMI	-.03	.05[b]	[-.17;.12][a]	.506[a]	-.03	-.60	.550					
Timeline / Chronicity	.52	.70[b]	[-1.90;2.67][a]	.460[a]	.04	.81	.422					
Cycle	.85	.67[b]	[-1.20;3.09][a]	.209[a]	.06	1.33	.186					
Consequences	1.95	.79[b]	[-.31;4.29][a]	.014[a]	.15	2.90	.004					
Personal Control	1.16	.72[b]	[-1.17;3.49][a]	.109[a]	.08	1.67	.095					
Treatment Control	-.46	.75[b]	[-2.79;2.16][a]	.530[a]	-.03	-.65	.520					
Coherence	-.26	.75[b]	[-2.76;2.13][a]	.731[a]	-.02	-.37	.712					
Emotional Representation	6.16	.66[b]	[4.04;8.20][a]	<.001[a]	.50	9.90*	<.001					
Gender	-4.03	1.44	[-9.80;.40][a]	.006[a]	-.14	-3.23*	.001					

Note. N = 397, * = significant (p < .002; see Bonferroni adjustment); BCA 99.8% CI; [1]values after bootstrapping; [a]based on 1,480 samples.

Summary of Hierarchical Regression Analysis for variables associated with ***Body Mass Index****.*

Variable	B	SE[1]	BCa[1]	p[1]	β	t	p	R	R^2	ΔR^2	F (df1,df2)	p (F change)
**Step 1 (control variables)**								.15	.02	.02*	9.65 (1,425)	.002
Age	-.13	.04[a]	[-.24;-.01][a]	.002[a]	-.150	-3.11*	.002					
**Step 2 (SIRs)**								.49	.24	.22*	16.39 (7,418)	<.001
Age	-.10	.03[b]	[-.21;0][b]	.003[b]	-.12	-2.80	.005					
Timeline / Chronicity	2.72	.50[b]	[1.10;4.28][b]	<.001[b]	.23	4.89*	<.001					
Cycle	1.04	.51[b]	[-.76;2.62][b]	.041[b]	.09	1.81	.071					
Consequences	3.11	.69[b]	[.80;5.41][b]	<.001[b]	.29	5.47*	<.001					
Personal Control	-1.42	.61[b]	[-3.11;.63][b]	.024[b]	-.12	-2.29	.022					
Treatment Control	1.39	.65[b]	[-1.15;3.67][b]	.029[b]	.11	2.20	.029					
Coherence	1.19	.63[b]	[-1.01;3.11][b]	.053[b]	.09	1.89	.059					
Emotional Representation	-.18	.58[b]	[-2.12;1.78][b]	.751[b]	-.02	-.33	.739					
**Step 3 (Gender)**								.50	.25	.01	15.09 (1,417)	.051
Age	-.09	.04[b]	[-.19;.03][b]	.014[b]	-.10	-2.23	.026					
Timeline / Chronicity	2.67	.50[b]	[1.06;4.16][b]	<.001[b]	.23	4.82*	<.001					
Cycle	.94	.51[b]	[-.81;2.54][b]	.064[b]	.08	1.62	.106					
Consequences	3.19	.69[b]	[.90;5.44][b]	<.001[b]	.30	5.61	<.001					
Personal Control	-1.27	.62[b]	[-3.04;.95][b]	.045[b]	-.11	-2.06	.041					
Treatment Control	1.35	.65[b]	[-1.11;3.52][b]	.032[b]	.10	2.15	.032					
Coherence	1.08	.62[b]	[-1.04;2.90][b]	.081[b]	.08	1.72	.086					
Emotional Representation	-.36	.59[b]	[-2.39;1.68][b]	.543[b]	-.04	-.66	.513					
Gender	-2.13	1.15[b]	[-5.60;1.10][b]	.064[b]	-.09	-1.96	.051					

Note. N = 427, * = significant ($p < .002$; see Bonferroni-Adjustment); BCA 99.8 % CI; [1]values after bootstrapping; [a]based on 1912 samples; [b]based on 1911 samples

Summary of Hierarchical Regression Analysis for variables associated with ***Restraint Eating Behavior****.*

**Variable**	**B**	**SE**	**β**	**t**	**p**	**R**	**R²**	**ΔR²**	**F (df1,df2)**	**p (F change)**
**Step 1 (control variables)**						.04	.00	.00	.36 (2,408)	.695
Age	.02	.03	.03	.52	.606					
BMI	.03	.04	.04	.75	.456					
**Step 2 (SIRs)**						.25	.06	.06	2.89 (7,401)	.001
Age	.04	.03	.07	1.34	.180					
BMI	-.00	.04	-.01	-.11	.916					
Timeline / Chronicity	-1.05	.49	-.12	-.2.15	.032					
Cycle	1.07	.49	.12	2.17	.031					
Consequences	.37	.51	.05	.73	.465					
Personal Control	-.57	.53	-.06	-1.09	.279					
Treatment Control	.95	.54	.10	1.76	.079					
Coherence	.22	.53	.02	.40	.687					
Emotional Representation	.62	.47	.08	1.34	.180					
**Step 3 (Gender)**						.31	.10	.03	4.19 (1,400)	<.001
Age	.08	.03	.12	2.29	.022					
BMI	-.02	.04	-.03	-.45	.651					
Timeline / Chronicity	-1.11	.48	-.13	-2.31	.021					
Cycle	.93	.49	.11	1.91	.057					
Consequences	.53	.50	.07	1.06	.290					
Personal Control	-.35	.52	-.04	-.67	.501					
Treatment Control	.89	.53	.09	1.68	.094					
Coherence	.03	.53	.00	.06	.953					
Emotional Representation	.33	.46	.05	.71	.478					
Gender	-3.56	.92	-.20	-3.87*	<.001					

Note. $N = 411$, * = significant ($p < .002$; see Bonferroni adjustment)

Summary of Hierarchical Regression Analysis for variables associated with ***Emotional Eating Behavior****.*

**Variable**	**B**	**SE**	**β**	**t**	**p**	**R**	**R²**	**ΔR²**	**F (df1,df2)**	**p (F change)**
**Step 1 (control variables)**						.16	.02	.02	5.07 (2,408)	.007
Age	-.11	.05	-.12	-2.43	.015					
BMI	.09	.05	.08	1.68	.093					
**Step 2 (SIRs)**						.46	.21	.19*	11.83 (7,401)	<.001
Age	-.09	.04	-.10	-2.06	.041					
BMI	-.08	.06	-.07	-1.39	.166					
Timeline / Chronicity	2.59	.69	.20	3.93*	<.001					
Cycle	.61	.66	.05	.92	.360					
Consequences	1.30	.69	.11	1.89	.059					
Personal Control	2.01	.71	.15	2.86	.005					
Treatment Control	-.81	.72	-.06	-1.13	.261					
Coherence	2.45	.72	.17	3.41*	.001					
Emotional Representation	2.64	.62	.24	4.23*	<.001					
**Step 3 (Gender)**						.49	.24	.03*	12.58 (1,400)	<.001
Age	-.05	.04	-.05	-1.03	.302					
BMI	-.10	.06	-.09	-1.76	.080					
Timeline / Chronicity	2.50	.65	.19	3.87*	<.001					
Cycle	.42	.65	.03	.66	.526					
Consequences	1.52	.68	.13	2.24	.025					
Personal Control	2.32	.70	.18	3.32*	.001					
Treatment Control	-.89	.71	-.06	-1.26	.209					
Coherence	2.20	.71	.15	3.11*	.002					
Emotional Representation	2.24	.62	.21	3.60*	<.001					
Gender	-4.88	1.23	-.19	-3.94*	<.001					

Note. $N = 411$, * = significant ($p < .002$; see Bonferroni adjustment)

Summary of Hierarchical Regression Analysis for variables associated with ***External Eating Behavior****.*

Variable	B	SE	β	t	p	R	R²	ΔR²	F (df1,df2)	p (F change)
**Step 1 (control variables)**						.22	.05	.05*	10.53 (2,408)	<.001
Age	-.16	.04	-.22	-4.56*	<.001					
BMI	-.01	.04	-.01	-.12	.902					
**Step 2 (SIRs)**						.46	.21	.17*	12.14 (7,401)	<.001
Age	-.15	.03	-.21	-4.48*	<.001					
BMI	-.12	.04	-.14	-2.79	.006					
Timeline / Chronicity	2.25	.52	.22	4.34*	<.001					
Cycle	.35	.52	.03	.66	.508					
Consequences	.97	.54	.11	1.80	.073					
Personal Control	2.25	.56	.22	4.05*	<.001					
Treatment Control	-.66	.57	-.06	-1.15	.251					
Coherence	.89	.57	.08	1.57	.118					
Emotional Representation	1.91	.49	.22	3.89*	<.001					
**Step 3 (Gender)**						.47	.23	.01	11.62 (1,400)	.018
Age	-.13	.04	-.18	-3.76*	<.001					
BMI	-.13	.04	-.15	-3.01	.003					
Timeline / Chronicity	2.21	.52	.22	4.28*	<.001					
Cycle	.25	.52	.03	.49	.627					
Consequences	1.08	.54	.12	2.00	.046					
Personal Control	2.40	.56	.23	4.31*	<.001					
Treatment Control	-.69	.57	-.06	-1.23	.221					
Coherence	.77	.57	.07	1.36	.176					
Emotional Representation	1.72	.50	.20	3.47*	.001					
Gender	-2.34	.99	-.12	-2.38	.018					

Note. $N = 411$, * = significant ($p < .002$; see Bonferroni adjustment)
